# Comparison of Diagnostic Performance of Ultrasonography and Magnetic Resonance Enterography in the Assessment of Active Bowel Lesions in Patients with Crohn’s Disease: A Systematic Review and Meta-Analysis

**DOI:** 10.3390/diagnostics12082008

**Published:** 2022-08-19

**Authors:** Da In Lee, Myung-Won You, So Hyun Park, Mirinae Seo, Seong Jin Park

**Affiliations:** 1 Department of Radiology, Kyung Hee University Hospital, Seoul 02447, Korea; 2 Department of Radiology, Gil Medical Center, Gachon University College of Medicine, Incheon 21936, Korea

**Keywords:** Crohn’s disease, ultrasound, magnetic resonance enterography, diagnostic performance, meta-analysis, disease activity

## Abstract

We aimed to evaluate and compare the diagnostic performances of ultrasonography (US) and magnetic resonance enterography (MRE) in assessing active bowel lesions in patients with Crohn’s disease (CD). Materials and Methods: We searched PubMed and EMBASE for studies in which US and MRE were used to assess active bowel lesions in CD patients. Bivariate random effect meta-analytic methods were used to estimate pooled sensitivity, specificity, and hierarchical summary receiver operating characteristic (HSROC) curves. We performed a meta-regression analysis to explore the source of study heterogeneity. Results: Eleven studies involving 752 patients were included. US exhibited a pooled sensitivity of 86% (95% confidence interval (CI) 72–94), pooled specificity of 88% (95% CI 78–94), and HSROC of 0.93 in 10 studies. MRE exhibited a pooled sensitivity of 88% (95% CI 76–95), pooled specificity of 87% (95% CI 73–95), and an HSROC of 0.94 in eight studies. In seven studies comparing the diagnostic performances of US and MRE, the summary sensitivity of US and MRE were 86% (95% CI 65–96, I^2^ = 92.1) and 86% (95% CI 72–93, I^2^ = 88.1) (*p* = 0.841), respectively. The summary specificity of US and MRE were 87% (95% CI 78–93, I^2^ = 79.8%) and 84% (72–90, I^2^ = 72.5%) (*p* = 0.431), respectively, which showed no statistical differences. On meta-regression analysis, studies from Europe (*p* = 0.002), those that used linear US probes (*p* = 0.012), those on small bowel lesions (*p* = 0.01), and those with outcomes as combined features (active inflammation) reported higher US sensitivity than those from other regions, those that used both linear and convex US probes, those on small and large bowels, and those with outcome as one feature (bowel wall thickening or ulcer). Studies with pediatric patients (*p* = 0.001), those with reference standards including US (*p* = 0.001), and outcomes as combined features (*p* = 0.01) reported higher MRE specificity than those with adult populations, reference standards other than the US, and outcomes as one feature. Conclusions: In spite of considerable heterogeneity in the included studies, both US and MRE can diagnose active bowel lesions with comparable diagnostic accuracy in patients with CD. The study region, type of US probe, lesion location, investigated outcome for US sensitivity and study population, reference standards, and investigated outcomes for MRE specificity were potential sources of heterogeneity.

## 1. Introduction

Crohn’s disease (CD) is a chronic inflammatory disease with a remitting and relapsing course, characterized by transmural inflammation of the gastrointestinal tract. Cross-sectional imaging methods, such as computed tomography (CT) and magnetic resonance imaging (MRI), are increasingly used for the initial diagnosis of active lesions, determining the distribution of the disease, detecting complications of the disease, and follow-up after treatment, especially for small bowel lesions that cannot be accessed by standard colonoscopy [1]. Although CT enterography (CTE) has become a standard imaging tool for CD evaluation, potential ionizing radiation risks are associated with the repeated CT scans that are performed over the chronic course of the disease [2]. In this context, magnetic resonance enterography (MRE) has been developed as an alternative radiation-free imaging technique for CTE and has largely replaced CTE as the primary cross-sectional imaging modality for both adult and pediatric patients with CD. Although both CTE and MRE have demonstrated similar diagnostic abilities for diagnosing active inflammation of the bowel [3,4], MRE is often preferred to CTE because of the absence of ionizing radiation, very high soft tissue contrast, and lower incidence of adverse events [5]. Furthermore, MRE is the most validated imaging modality for disease evaluation and therapeutic monitoring in the research field [5,6]

Intestinal ultrasound (US) has recently gained attention for its use in evaluating active or relapsed bowel lesions, severity of inflammation, and monitoring therapeutic responses. It can be a useful alternative tool to CTE in terms of several advantages, as it is a noninvasive radiation-free technique that is well tolerated and easy to repeat [7]. Hence, both US and MRE are preferred over CT in diagnosing CD lesions, particularly in young patients. Previous studies reported that US demonstrated higher accuracy in diagnosing active lesions in the terminal ileum and colon than MRE [8,9]. However, diagnostic accuracy of MR exam without luminal distention using oral contrast media was compared with that of US exam in those studies. To our knowledge, there are scanty data regarding comparison between US and MRE in the assessment of active CD lesion. Therefore, in this meta-analysis, we investigated and compared the diagnostic performance of US and MRE in the diagnosis of active bowel inflammation in patients with CD.

## 2. Materials and Methods

This meta-analysis followed the guidelines of the Preferred Reporting Items for Systematic Reviews and Meta-analyses for Diagnostic Test Accuracy Studies (PRISMA-DTA) statement [10], and the analytic methods recommended by the Diagnostic Test Accuracy Working Group of the Cochrane Collaboration [11] and the Agency for Healthcare Research and Quality (AHRQ) [12].

### 2.1. Data Sources

A thorough search of the PubMed MEDLINE and EMBASE databases was conducted to identify original research articles that investigated the diagnostic performance of US and MRE in diagnosing active bowel lesions or disease activity in patients with CD. The search query was developed to provide a sensitive literature search to avoid missing relevant articles. We then manually evaluated the searched articles to further narrow down the number of relevant articles. The following search terms were used: (Crohn OR Crohn’s) AND (“magnetic resonance enterography” OR MRE OR “MR enterography”) AND (ultrasound OR ultrasonography OR US) AND (“diagnostic performance” OR “diagnostic accuracy” OR “accuracy”). No beginning-date limit was used, and we continued updating the literature search until January 2022. Additionally, our search was restricted to human subjects and English-language studies. To expand the search, bibliographies of articles that survived the selection process were screened for other potentially suitable articles. Endnote version x20 (Thomson Reuters, New York, NY, USA) was used to manage the literature.

### 2.2. Study Selection

After removing duplicate articles, the articles were reviewed for eligibility: (1) population, patients with CD; (2) index test, US or MRE; (3) comparator test, MRE or US; (4) outcomes, sensitivity, and specificity in the dichotomous diagnosis of active bowel inflammation (i.e., presence or absence of active bowel lesion/bowel wall thickening/ulcer lesion, etc.); and (5) study design, observational studies (retrospective or prospective), and clinical trials. We included studies that investigated and compared the diagnostic performance of both US and MRE; however, we also included studies that investigated either the US or MRE and used the other modality as a reference standard, although no comparison between US and MRE was performed. The exclusion criteria were as follows: (1) case reports, review articles, editorials, letters, comments, and conference abstracts/proceedings; (2) studies that were not within the field of interest of this study; (3) studies with overlapping patients and data (in such cases, the duplicated data were included only once in this study); and (4) studies with insufficient details to construct a diagnostic 2-by-2 table of the imaging results of active bowel lesions. The articles were screened based on their titles and abstracts. The full text of the articles was reviewed after selecting potentially eligible abstracts. Both steps were performed by two independent reviewers and disagreements were resolved through discussion.

### 2.3. Data Extraction and Quality Assessment

The following data were extracted onto a predefined data form: (1) study characteristics, including authors, year of publication, hospital or medical school, region where the study was conducted, time and period of patient recruitment, and study design (prospective versus retrospective); (2) study population characteristics, including total number of included patients, total number of studied bowel segments (in studies with a mention of the same), age group (adults versus pediatrics), and status of CD (confirmed disease versus suspected state); (3) index test and its technique, including imaging modality whether US or MRE, type of equipment, number and kind of US probe, and usage of special technique, such as small intestine oral contrast US (SICUS) or contrast-enhanced US (CEUS); (4) comparator test and its technique, including imaging modality whether US or MRE, magnetic resonance (MR) scanner field strength, and scanning protocol; (5) details of reference standards and the time interval between index/comparator and reference standards; (6) blinding to reference standards when interpretating results of index/comparator tests; and (7) study outcomes of the accuracy of the dichotomous diagnosis of active bowel lesion and method of outcome analysis (per patient versus per segment). To determine diagnostic accuracy, the exact numbers of true positives, true negatives, false positives, and false negatives of active bowel lesions determined by either US or MRE were extracted.

We used the Quality Assessment of Diagnostic Accuracy Studies (QUADAS-2) criteria [13] to assess the quality of the selected articles. The QUADAS-2 tool assesses study quality in four domains: patient selection, index test, reference standard, and flow of patients through the study and timing of the index test and reference standard. For each article, the risk of bias was assessed for each of the four domains and applicability, that is, generalizability was determined for each of the first three domains [13]. Two reviewers independently performed data extraction and quality assessment, and any discrepancies were resolved through discussion.

### 2.4. Data Synthesis and Analysis

We used a bivariate random-effects model to analyze and pool the diagnostic performance (sensitivity and specificity) measurements across studies. To derive summary estimates of diagnostic performance, we plotted estimates of the observed sensitivities and specificities for each test in forest plots and hierarchical summary receiver operating characteristic (HSROC) curves derived from individual study results [14,15]. These results were plotted using HSROC curves with 95% confidence and prediction regions. Heterogeneity was determined using the Cochran Q test (*p* < 0.05, indicating the presence of heterogeneity) and Higgins I^2^ statistics; and I^2^ > 50% was considered to indicate the substantial heterogeneity [16] When heterogeneity was noted, heterogeneity by threshold effect was analyzed through visual assessment of the coupled forest plots of sensitivity and specificity, as well as by calculating the Spearman correlation coefficient between the sensitivity and false-positive rate (i.e., 1-specificity). A correlation coefficient >0.6 was considered to indicate a considerable threshold effect. Deeks’ funnel plots of individual studies to check for publication bias were omitted according to the revised PRISMA-DTA.

### 2.5. Meta-Regression Analyses

A meta-regression analysis was performed to further explore the causes of study heterogeneity by including covariates in the bivariate model. We considered the following covariates: (1) study design (retrospective versus prospective); (2) total number of included patients (≥30 versus <30); (3) region where the study was conducted (Europe versus other regions); (4) study population (adult versus pediatric); (5) CD status (confirmed CD versus including suspected CD); (6) blinding to reference standard while interpreting index and comparator test results (blinded versus not mentioned); (7) reference standards (including MRE versus other methods/including US versus other methods); (8) type of US (conventional US versus US with a special technique, such as SICUS or CEUS); (9) type of US probes (both linear and convex versus linear alone); (10) method of outcome analysis (per patient versus per segment); (11) lesion location (small bowel versus small and large bowels); and (12) outcome (combined imaging features as active inflammation versus one imaging feature as ulcer or bowel wall thickening).

We used the mada package in R software version 3.4.1 (R Foundation for Statistical Computing, Vienna, Austria) and the midas and metandi modules in STATA 17 (StataCorp LP, College Station, TX, USA) for statistical analysis, with *p* < 0.05 indicating statistical significance.

## 3. Results

### 3.1. Literature Search

**Figure 1:**
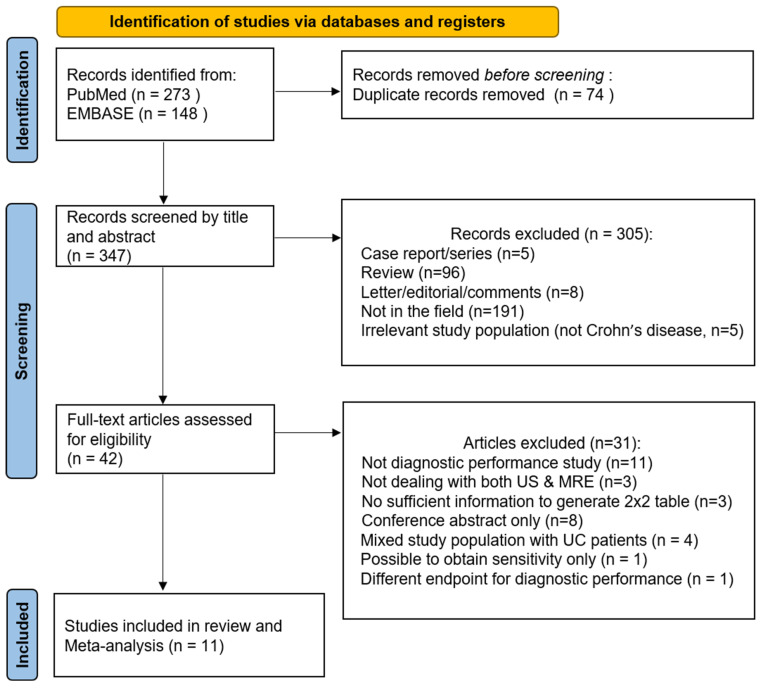
Study selection flow diagram.

Following the removal of duplicate records, 305 records were excluded during screening by title and abstract. The remaining 42 articles were assessed for eligibility with full text and 31 articles were excluded for the following reasons: (1) no evaluation of diagnostic performance (i.e., evaluation of the correlation between US and MRE, *n* = 11), (2) no evaluation of both US and MRE (did not consider both US and MRE as imaging modality or reference standard, *n* = 3), (3) insufficient information to generate a two-by-two table (*n* = 3), (4) mixed study population with patients with ulcerative colitis (*n* = 4), (5) conference abstract only and no full text available (*n* = 8), (6) study presenting sensitivity results alone (*n* = 1), and (7) different endpoints for diagnostic performance (i.e., stricture, *n* = 1). Finally, 11 studies [17,18,19,20,21,22,23,24,25,26,27] comprising 752 patients evaluating the diagnostic performance of US and/or MRE for active bowel lesions were included (Figure 1).

### 3.2. Characteristics of the Studies and the Included Patients

Characteristics of the study design and population are listed in Table 1. A total of 10 of the 11 studies had a prospective design [17,18,19,21,22,23,24,25,26,27], while only one was a retrospective study [20] Furthermore, the majority of studies have been conducted in Europe [17,18,19,20,21,23,24,25,26]. The total number of enrolled patients ranged from 17 to 284. Most of the studies were investigated by using per-patient analyses, while three studies were investigated by using per-segment analyses, with the total number of included lesions ranging from 91 to 426 [18,23,27] Most studies were conducted on adult populations, except for three that were pediatric studies [18,20,23]. The status of CD was mostly confirmed, except two studies that involved suspected CD [18,22].

Table 2 presents the study characteristics with respect to the imaging techniques and diagnosis. In most of the studies, the index test was US except for one study [23]. As seven studies used MRE as comparators, we performed a direct comparison between the diagnostic performance of US and MRE in the seven studies by using the meta-analytic summary results [18,21,22,24,25,26,27]. Three studies evaluated the diagnostic performance of US using a kind of linear US probe [21,23,26], while the other eight studies used both linear and convex probes. The investigated lesion location was usually confined within small bowels [18,19,20,21,23,24,25,26]; in three studies, active lesions both in small and large bowels were evaluated. [17,22,27]. The outcome of most studies was the presence of active bowel inflammation [17,18,19,20,21,23,24,25], which was defined as a combination of imaging features, such as bowel wall thickening, loss of wall stratification, increased vascularity, and extramural findings or complications, although the specific criteria were slightly different among the studies. In one study, the outcome was to discriminate severe from mild active lesions by evaluating the severity of the disease [24]. Three studies investigated an imaging feature, such as ulcer with reference to the pathology [26] or bowel wall thickening with reference to colonoscopy [22,27], as an outcome for diagnostic performance evaluation, and we assumed that these outcomes could be interpreted as representing features of disease activity and included in this analysis. References in the included studies were mostly pathology or/and colonoscopy [17,18,21,22,23,24,26,27]. MRE was included as a reference standard in three studies [17,19,20], while US was used in one study [23].

### 3.3. Quality of Studies

**Figure 2:**
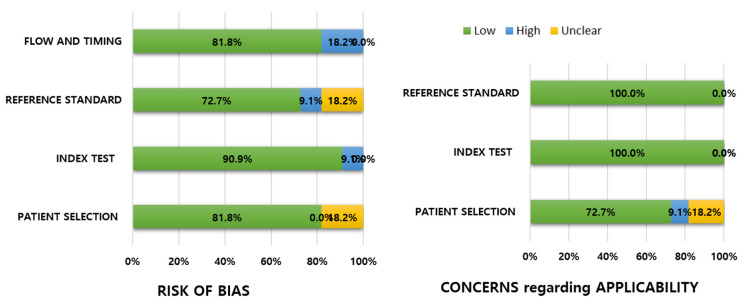
Grouped bar charts showing risk of bias and concern for applicability of 11 included studies assessed using Quality Assessment of Diagnostic Accuracy Studies-2 domains.

The quality of the included studies is summarized in Figure 2. Major concerns regarding the risk of bias were uncertainty about consecutive patient selection [20,22], unclear blinding of reference standards when interpreting the index test [20,22], uncertain independency between the reviewers or the same reviewers for the index test and reference test [19], nonacceptable interval between the index test and reference standards (long intervals between the two exams) [20], different reference standards among the included patients, or only a partial section of patients received the intended reference exam. [20]. Mostly, no significant concerns regarding the applicability were observed, except for few concerns on patient selection because the study population included not only confirmed CD, but also suspected CD [18,22], and unclear diagnostic criteria of CD in a retrospective study [20].

### 3.4. Diagnostic Performance of US

**Figure 3:**
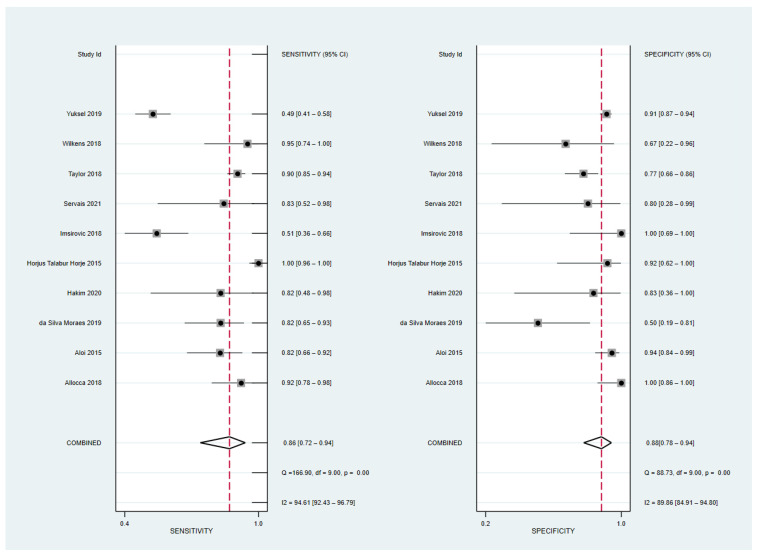
Coupled forest plots of overall pooled sensitivity and specificity for diagnosing active bowel lesions in patients with Crohn’s disease using ultrasonography [17,18,19,20,21,22,24,25,26,27].

The sensitivity and specificity of the 10 included studies with 595 patients [17,19,20,21,22,24,25,26] and 517 bowel segments [18,27] ranged from 51 to 100 and 50 to 100, respectively. The pooled sensitivity and specificity were 86% (95% CI, 72–94%) and 88% (95% CI, 78–94%), respectively, and both the Q test (Q = 62.97, *p* = 0.0001) and Higgins I^2^ statistics demonstrated a considerable study heterogeneity, both in the sensitivity (I^2^ = 94.61%) and specificity (I^2^ = 89.86%). A threshold effect was absent not only from the visual analysis of the coupled forest plot of sensitivity and specificity (Figure 3), but also from the corresponding correlation coefficient of −0.20 between the sensitivity and false-positive rate. The area under the HSROC curve was 0.93 (95% CI 0.91–0.95), and the 95% confidence and prediction regions revealed a large difference between the two regions, indicating considerable heterogeneity between studies.

### 3.5. Diagnostic Performance of MRE

**Figure 4:**
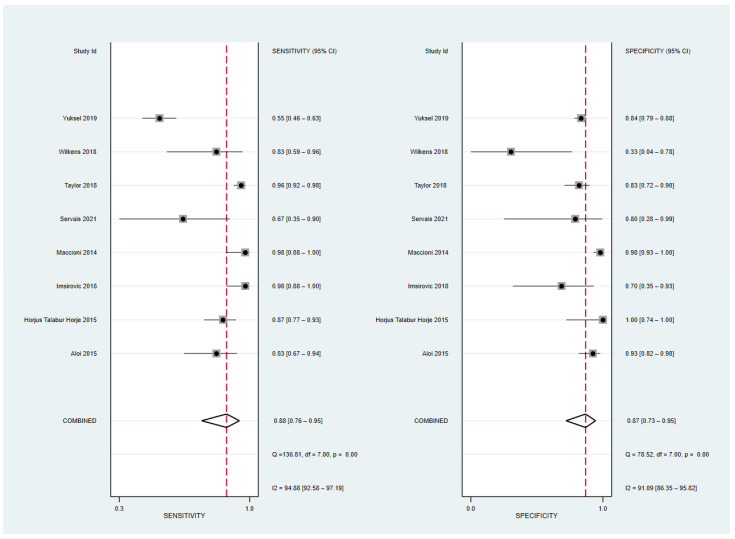
Coupled forest plots of the overall pooled sensitivity and specificity for diagnosing active bowel lesions in patients with Crohn’s disease using MR enterography. MR, magnetic resonance [18,21,22,23,24,25,26,27].

**Figure 5:**
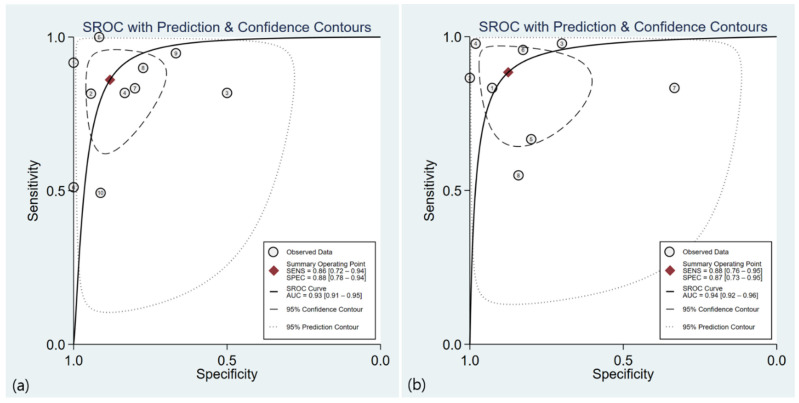
Hierarchical summary receiver operating characteristic (HSROC) curve of the diagnostic performance of (**a**) US and (**b**) MRE for diagnosing active bowel lesions in patients with Crohn’s disease. US, ultrasonography; MRE, magnetic resonance enterography.

The sensitivity and specificity of the eight included studies with 474 patients [21,22,24,25,26] and 667 bowel segments [18,23,27] ranged from 54.9 to 97.8 and 33.3 to 100, respectively. The pooled sensitivity and specificity were 88% (95% CI, 76–95%) and 87% (95% CI, 73–95%), respectively (Figure 4), and both the Q test (Q = 33.68, *p* = 0.001) and I^2^ statistics revealed a considerable heterogeneity, both in the sensitivity (I^2^ = 94.88) and specificity (I^2^ = 91.09). A threshold effect was absent, either by visual analysis of the forest plot or from the corresponding correlation coefficient of 0.23. The area under the HSROC curve was 0.94 (95% CI, 0.91–0.96) (Figure 5). The Deeks’ funnel plot and asymmetry test (*p* = 0.27 for the slope coefficient) both indicated no influence of publication bias on our meta-analysis.

### 3.6. Meta-Regression Analyses

Meta-regression analysis was performed to investigate the potential sources of heterogeneity in the US (Table 3) and MRE results (Table 4). The study region, type of US probe, lesion location, and definition of outcome were significant factors for the heterogeneity of sensitivity; that is, studies conducted in Europe compared to those in other regions (*p* = 0.002), studies which used only linear US probes compared to those using both linear and convex US probes (*p* = 0.01), studies that investigated active inflammation as an outcome, which was defined with combined imaging features compared to those that investigated outcomes defined as one imaging feature (ulcer or bowel wall thickening) (*p* = 0.01), and studies that investigated lesions confined within the small bowel (*p* = 0.01) compared to those that investigated lesions located both in small and large bowels reported higher sensitivities. No significant potential source of heterogeneity was observed for the specificity of the US examination.

Regarding MRE results, study population, reference standards, and definition of outcome were significant factors for the heterogeneity of specificity; that is, higher specificities were reported by studies conducted on pediatric patients than those on adult patients (*p* = 0.0002), studies with reference standards including US examination than those with other forms of reference standards (*p* = 0.001), and studies which investigated active inflammation as an outcome than those that investigated a single imaging feature, such as ulcer or bowel wall thickening as an outcome (*p* = 0.01).

### 3.7. Comparison of Diagnostic Performance of US and MRE in Diagnosing Active Bowel Lesions

We performed a subgroup analysis and direct comparison of the diagnostic ability of US and MRE using the random effects model in seven studies including 475 patients [21,22,24,25,26] and 517 bowel segments [18,27] (Table 5). The pooled sensitivities of US and MRE were 86% (95% CI, 65.3–96.6%) and 86% (95% CI, 72.4–93.6%), respectively, with considerable heterogeneity (I^2^ = 92.1% and 88.1%). The pooled specificity of US and MRE was 87% (95% CI, 78–93.1%) and 84% (95% CI, 72.5–90.7%), respectively, with substantial heterogeneity, both in US results (I^2^ = 79.8%) and MRE results (I^2^ = 72.5%).

## 4. Discussion

This study demonstrated the diagnostic performance of US and MRE for active bowel inflammation to be similar in both the indirect comparison of meta-analytic summary results and direct comparison in the subgroup analysis. Considerable heterogeneity was observed in the pooled sensitivity and specificity of both US and MRE; therefore, we performed a meta-regression analysis with potential sources of study heterogeneity. The study region (Europe > other regions), type of US probe used (linear > both linear and convex), lesion location (small bowel > small and large bowels), and definition of outcome (active inflammation > ulcer or bowel wall thickening) were the significant factors for heterogeneity in US sensitivity, and the study population (pediatrics > adults), reference standards (including US > others), and definition of outcome (active inflammation > ulcer or bowel wall thickening) were the significant factors for heterogeneity in MRE specificity.

In a previous meta-analysis on the diagnostic performance of US in detecting active CD, the pooled sensitivity and specificity were 88% and 97%, respectively, which are slightly higher than observed in our results (87%/87%) [28]. Lesion location was the source of heterogeneity, indicating that patients with disease located in the small bowel had a higher diagnostic odds ratio (DOR) than those with disease located in the colon or those with an unclear status (332.46 vs. 60.24); these findings were consistent with our study results. Based on the previous meta-analyses on the diagnostic performance of MRE in diagnosing active CD, our study and the study by Ahmed et al. reported similar results on the diagnostic accuracy of MRE in the diagnosis of active small bowel lesions (88%/88%, 88%/87%) [29] Furthermore, although they included studies with lesions located in both small and large bowels, only studies with per-patient analysis in the pooling of diagnostic accuracy were included, while we included studies with both per-patient and per-segment analyses. On the other hand, a meta-analysis on the diagnostic accuracy of MRE in the diagnosis of active inflammation in the colon reported lower pooled sensitivity and higher pooled specificity (69%/95%) compared with that reported by Ahmed et al. and in our study [30]. In the subgroup analysis in this study, MRE sensitivity slightly increased in pediatric patients (80%/95%) compared with those in adults (62%/94%). Another study presented a similar diagnostic accuracy of MRE (84%/97%) for the diagnosis of active small bowel lesions in pediatrics [31]. In those studies, the diagnostic accuracy of MRE in pediatric patients exhibited superior MRE specificity to our meta-analysis, which is consistent with our meta-regression analysis results that revealed that studies with pediatric patients had a significantly higher MR specificity than studies with adults.

Both US and MRE are the main tools available for the frequent reassessment required by the relapsing nature of CD and the relatively young age at which most patients are diagnosed. MRE and US are preferable radiation-free techniques and are useful for assessing disease activity, extent, and extramural complications [32]. “Active bowel inflammation” can be diagnosed with a combination of various imaging features, including bowel wall thickening ≥ 3 mm, segmental mural hyperenhancement, stricture, ulceration, sacculation for bowel wall along with perienteric edema/inflammation, engorged vasa arecta, fibrofatty proliferation, lymphadenopathy, diminished motility, and presence of penetrating complications, such as sinus tract, fistula, and inflammatory mass or abscess common to both modalities [33] Additional features for active CD lesions are intramural edema and restricted diffusion on MRE and increased color Doppler flow on US [34]

For comparison of diagnostic performance of the two modalities, Panes et al. reported the pooled sensitivity and specificity of US and MR for assessment of disease activity to be 85% and 91%, and 80% and 82%, respectively [35]. The diagnostic accuracy of US was higher than that of MR in the diagnosis of active bowel lesions in the terminal ileum and colon [8,9]; however, proper luminal distention with oral contrast media was not used in those studies, producing more false-negative results in MR examination. Kopylov et al. studied the diagnostic performance of SICUS and MRE compared with capsule endoscopy (CE) in the diagnosis of active small bowel lesions and reported a similar DOR for SICUS (0.88, 95% CI 0.51–1.53) and MRE (1.17, 95% CI 0.83–1.67) [36]. However, MRE showed significantly lower diagnostic yield compared with CE for active lesions in the proximal small bowel (OR 2.79, 95% CI 1.2–6.48), while US showed no significant difference from CE (OR 2.76, 95% CI 0.84–9.02) [36]. In our study, diagnostic accuracy of MRE was slightly higher than that of US (HSROC 0.94 vs. 0.93); however, no statistical differences were observed in both indirect and direct comparisons. On the other hand, the sensitivity of US for active small bowel lesions was higher than that for active lesions in both small and large bowels, which is consistent with the results of Kopylov et al. Although MRE did not exhibit significant differences in specificity according to lesion location, the joint model of sensitivity and specificity presented higher diagnostic accuracy with active small bowel lesions than with active lesions in both the small and large bowels (*p* = 0.02, not shown). Both US and MRE are more beneficial for the assessment of the small bowel, where standard endoscopy is not fully accessible.

Generally, MRE has a limitation in detecting subtle mucosal lesions but can adequately assess extraparietal involvement, while US can demonstrate the structure of the bowel wall and mucosal lesions more clearly but with limited general assessment of whole bowel lesions [37]. According to our meta-regression analysis, active bowel inflammation can be more accurately diagnosed with a combination of imaging features than with a single imaging feature, such as bowel wall thickening or ulcer lesion on both modalities.

Studies with SICUS or CEUS were included (three studies) and analyzed to determine whether they were better than the conventional US. Previous studies reported superior sensitivity in the detection of small bowel lesions with SICUS (96–100% vs. 57–96%) and reduced interobserver variability compared with the conventional US [38]. Likewise, the previous meta-analysis reported the pooled sensitivity, specificity, and summary AUC of CEUS for an active CD to be 93%, 87%, and 0.96, respectively, which were superior to those of US in our study [39]. Although the sensitivity and specificity of SICUS/CEUS were higher than those of conventional US, no significant differences were observed in our study.

Regarding the US probe, convex probes are used for panoramic evaluation and rectosigmoid colons, and linear probes are used for detailed examination of the bowel wall [40] Three studies solely using linear probes investigated the active bowel lesions focused on the small bowels [21,23,26] and demonstrated superior US sensitivity compared to those using both linear and convex probes. Therefore, US examination using a linear probe can provide sufficient high resolution for small-bowel imaging, regardless of the use of a convex probe.

This study has several limitations. First, a small number of studies were included, especially for direct comparison between US and MRE. Second, we included studies with both per-patient and per-segment analyses in the pooled analysis. However, this might prevent the overestimation of the sensitivity in the per-patient analysis. Third, the reference standards of the included studies were heterogeneous, ranging from pathology, colonoscopy, and clinical panel diagnosis to imaging modalities, such as MRE or the high-resolution US. Therefore, we performed a meta-regression analysis of heterogeneous references and there was no significant difference in accuracy of both US and MRE between the subgroups. Fourth, a small or unbalanced number of studies in the subgroup might have limited the evaluation of few variates in the meta-regression analysis.

In conclusion, although considerable heterogeneity was observed, both US and MRE demonstrated comparable diagnostic accuracies in the diagnosis of active bowel lesions in patients with CD. Considering several factors, for the study of heterogeneity affecting US sensitivity and MRE specificity, US examination for disease in the small bowel using a linear probe and MRE examination for pediatrics might be useful, while a combination of imaging features representing disease activity should be assessed for both US and MRE. With this consideration, we can make the most of the US and MRE for monitoring of disease activity and timely management.

## Figures and Tables

**Table 1:** Study characteristics of patients.

Author	Study Design	Study Region	No. of Patients (n)	No. of Lesions (n)	Method of Analyses	Study Population	Crohn’s Disease status
Allocca et al. (2018) [17]	Prospective	Milano, Italy	60		Per-patient	Adult > 18 y	Confirmed
Aloi et al. (2015) [18]	Prospective	Rome, Italy	25	91	Per-segment	Pediatrics	confirmed + suspected
da Silva Moraes et al. (2019) [19]	Prospective	Rio de Janeiro, Brazil	43		Per-patient	Adult	Confirmed
Hakim et al. (2020) [20]	Retrospective	London, UK	17		Per-patient	Pediatrics	Confirmed
Horjus et al. (2015) [21]	Prospective	Arnhem, Netherland	105		Per-patient	Adult	confirmed active
Imsirovic et al. (2018) [22]	Prospective	Bosnia and Herzegovina	55		Per-patient	Adult	Suspected
Maccioni et al. (2014) [23]	Prospective	Rome, Italy	50	150	Per-segment	Pediatrics	Confirmed
Servais et al. (2021) [24]	Prospective	Lyon, France	17		Per-patient	Adult > 18 y	Confirmed
Taylor et al. (2018) [25]	Prospective	London, UK	284		Per-patient	Adult > 16 y	Newly diagnosed + relapsed
Wilkens et al. (2018) [26]	Prospective	Silkeborg, Denmark	25		Per-patient	Adult > 18 y	Confirmed
Yuksel et al. (2019) [27]	Prospective	Ankara, Turkey	71	426	Per-segment	Adult	Confirmed

UK, United Kingdom.

**Table 2:** Study characteristics regarding imaging modalities.

Author	Index Test	US Probe	Comparator	MR Magnet T	Blinding	Reference Standards	Outcome Variable
Allocca 2018 [17]	US	1–5 MHz (convex), 4–8 MHz (linear)		1.5	yes	Colonoscopy + MRE	active disease in terminal ileum and colon
Aloi 2015 [18]	SICUS	3.5 MHz (convex), 5 MHz (linear)	MRE	1.5	yes	Panel consensus dx + Colonoscopy	active small bowel disease
da Silva Moraes 2019 [19]	US	10–12 MHz(linear), 5–10 MHz(convex)	Clinical HBI	1.5	yes	MRE	active ileal disease
Hakim 2020 [20]	SICUS	3–9 MHz (convex) 6–11 MHz (linear)		1.5	NR	MRE	active small bowel lesion
Horjus 2015 [21]	CEUS using Sonovue	7.5 MHz (linear)	MRE	1.5	yes	Colonoscopy	active bowel lesion in terminal ileum
Imsirovic 2018 [22]	US	3.5 MHz (convex), 7 MHz (linear)	MRE	1.5	NR	Colonoscopy + pathology	bowel wall thickening in terminal ileum and colon
Maccioni 2014 [23]	MRE	7.5 MHz (linear)		1.5	yes	HRUS+ colonoscopy	localization of active small bowel lesion
Servais 2021 [24]	US	14 MHz (linear) 6 MHz(convex)	MRE	1.5	yes	Pathology	severe active small bowel lesion
Taylor 2018 [25]	US	2–5 MHz(convex), >5 MHz (linear)	MRE	1.5/3	yes	Panel consensus dx	active small bowel disease
Wilkens 2018 [26]	US	9 L4 probe (linear)	MRE	1.5	yes	Pathology	ulcer in small bowel
Yuksel 2019 [27]	US	3.5–5.5 MHz 7–12 MHz (linear)	MRE	1.5	yes	Colonoscopy	bowel wall thickening in terminal ileum and colon

US, ultrasound; SICUS, small intestine contrast US; CEUS, contrast enhanced US; MRE, magnetic resonance enterography; HBI, Harvey-Bradshaw index; NR, not reported.

**Table 3:** Meta-regression analysis of the diagnostic performance of US in diagnosing active bowel lesions in patients with Crohn’s disease.

Covariate	Subgroup	No. of Studies	Meta-Analytic Summary Estimates			
			Sensitivity (95% CI)	*p* Value	Specificity (95% CI)	*p* Value
Study design	Prospective	9	86% (75,97)	0.63	88% (80,96)	0.66
	Retrospective	1	84% (44,100)		85% (50,100)	
Total no. of patients	≥30	6	85% (71,99)	0.94	89% (79,98)	0.56
	<30	4	88% (73,100)		87% (72,100)	
Study region	Europe	7	91% (85,97)	0.002 *	88% (76,94)	0.38
	Not Europe	3	61% (40,83)		85% (71,100)	
Study population	Adult	8	87% (76,98)	0.92	87% (77,96)	0.58
	Pediatrics	2	82% (68,90)		93% (83,97)	
Crohn’s disease status	Confirmed CD	7	90% (80,99)	0.14	85% (75,95)	0.38
	Including suspected CD	3	71% (50,86)		94% (86,98)	
Reference standards	Including MRE	3	87% (80,94)	0.82	85% (68,100)	0.99
	Others	7	86% (73,98)		89% (80,97)	
Type of US	Conventional US	7	83% (67,92)	0.26	85% (60,95)	0.36
	SICUS/CEUS	3	95% (60,100)		93% (84,97)	
Type of US probe	Both linear and convex	8	79% (68,90)	0.01 *	89% (81,97)	0.68
	Linear	2	99% (97,100)		84% (60,95)	
Method of outcome analysis	Per patient	8	90% (82,98)	0.11	83% (73,94)	0.97
	Per segment	2	67% (32,100)		93% (86,100)	
Lesion location	Small bowel	7	91% (85,98)	0.01 *	83% (71,94)	0.19
	Small and large bowels	3	67% (43,91)		96% (90,100)	
Outcome variable	Combined features	7	90% (84,97)	0.01 *	87% (77,97)	0.92
	One feature	3	67% (41,92)		90% (78,100)	

* means *p*-value < 0.05.

**Table 4:** Meta-regression analysis of the diagnostic performance of MRE in diagnosing active bowel lesions in patients with Crohn’s disease.

Covariate	Subgroup	No. of Studies	Meta-Analytic Summary Estimates			
			Sensitivity (95% CI)	*p* Value	Specificity (95% CI)	*p* Value
Total no. of patients	≥30	5	92% (76,98)	0.26	91% (77,97)	0.31
	<30	3	80% (69,88)		78% (39,95)	
Study region	Europe	6	90% (81,95)	0.62	90% (70,97)	0.41
	Not Europe	2	87% (32,99)		84% (79,87)	
Study population	Adult	6	87% (70,95)	0.46	83% (79,87)	0.0002 *
	Pediatrics	2	93% (74,98)		96% (91,99)	
Crohn’s disease status	Confirmed CD	6	87% (70,95)	0.45	88% (67,96)	0.89
	Including suspected CD	2	93% (74,98)		88% (67,96)	
Reference standards	Others	7	86% (76,96)	0.42	84% (80,88)	0.001 *
	Including US	1	98% (93,100)		98% (96,100)	
Type of US	Conventional US	6	90% (72,97)	0.68	83% (63,94)	0.17
	SICUS/CEUS	2	86% (78,91)		94% (85,98)	
Type of US probe	Both linear and convex	5	86% (66,96)	0.53	85% (81,88)	0.46
	Linear	3	90% (80,96)		94% (36,100)	
Method of Outcome analysis	Per patient	5	90% (80,99)	0.41	75% (61,90)	0.48
	Per segment	3	85% (68,100)		93% (88,99)	
Lesion location	Small bowel	6	90% (81,99)	0.61	90% (80,100)	0.45
	Small and large bowels	2	84% (62,100)		81% (54,100)	
Outcome	Combined features (active inflammation)	5	90% (81,99)	0.36	94% (88,99)	0.01 *
	One feature (bowel wall thickening/ulcer)	3	84% (67,100)		70% (47,93)	

US, ultrasound; CD, Crohn’s disease; CI, confidence interval; SICUS, small intestine contrast US; CEUS, contrast-enhanced US. * means *p*-value < 0.05.

**Table 5:** Direct comparison of diagnostic performance for active bowel lesions between US and MRE.

Author	US						MRE					
	TP	TN	FP	FN	Sensitivity (95% CI)	Specificity (95% CI)	TP	TN	FP	FN	Sensitivity (95% CI)	Specificity (95% CI)
Aloi et al. [18]	31	50	3	7	81% (65,92)	94% (84,98)	30	51	4	6	83% (67,93)	92% (82,98)
Horjus_Talabur et al. [21]	82	11	1	0	100% (95,100)	91% (61,99)	71	12	0	11	86% (77,93)	100% (73,100)
Imsirovic et al. [22]	23	10	0	22	51% (35,66)	100% (69,100)	44	7	3	1	97% (88,99)	70% (34,93)
Servais et al. [24]	10	4	1	2	83% (52,98)	80% (28,99)	8	4	1	4	66% (34,90)	80% (28,99)
Taylor et al. [25]	188	58	17	21	90% (85,93)	77% (66,86)	200	62	13	9	95% (92,98)	82% (72,90)
Wilkens et al. [26]	18	4	2	1	94% (74,99)	66% (22,95)	15	2	4	3	83% (58,96)	33% (43,77)
Yuksel et al. [27]	70	259	25	72	49% (40,57)	91% (87,94)	78	239	45	64	54% (46,63)	84% (79,88)
Higgins I^2^ for study heterogeneity					92.1%	79.8%					88.1%	72.5%
Summary estimate using the bivariate model					86% ^a^ (65,96)	87% ^b^ (78,93)					86% ^a^ (72,93)	84% ^b^ (72,90)

^a^ No significant difference in pooled sensitivities for diagnosing active bowel lesions between US and MRE, *p* = 0.84. ^b^ No significant difference in pooled specificities for diagnosing active bowel lesions between US and MRE, *p* = 0.43. US, ultrasound; MRE, magnetic resonance enterography; CI, confidence interval; TP, true positive; TN, true negative; FP, false positive; FN, false negative.

## Data Availability

No new data were created or analyzed in this study. Data sharing is not applicable to this article.
